# Design of 3‐aminophenol‐grafted polymer‐modified zinc sulphide nanoparticles as drug delivery system

**DOI:** 10.1049/nbt2.12063

**Published:** 2021-06-11

**Authors:** Milad Abniki, Zahra Azizi, Homayon Ahmad Panahi

**Affiliations:** ^1^ Department of Chemistry, Karaj Branch Islamic Azad University Karaj Iran; ^2^ Department of Chemistry, Central Tehran Branch Islamic Azad University Tehran Iran

## Abstract

Zinc sulphide (ZnS) nanoparticles were synthesized by the coprecipitation method. The ZnS nanoparticle surface was polymerized with allyl glycidyl ether (AGE), and 3‐aminophenol was then deposited as a ligand on nanosorbent. The modified nanosorbent was investigated with Fourier transform infrared spectroscopy and thermogravimetric analysis. The particle size of the modified nanosorbent was studied with scanning electron microscopy. Some characteristic factors of the adsorption process such as pH and time were investigated for famotidine using the modified nanosorbent. The equilibrium adsorption study of famotidine by 3‐aminophenol‐grafted AGE/ZnS was analysed by adsorption isotherms of the Langmuir, Freundlich, and Temkin models. The famotidine‐releasing process was investigated in simulated biological fluids (intestinal fluid at pH of 7.4 and gastric fluid at pH of 1.2) and demonstrated 65% and 73% famotidine release during periods of 30 h (pH = 7.4) and 60 min (pH = 1.2), respectively. These results reveal the optimal performance of 3‐aminophenol‐grafted AGE/ZnS for sustained drug delivery.

## 1 INTRODUCTION

In recent years, scientists have worked to increase the bioavailability of drugs in the body to improve effectiveness and reduce side effects. The human body's 50% to 60% water content interacts with drugs, thus influencing their bioavailability. Low water solubility causes some drugs to have a lower absorption volume in the gastrointestinal tract. This lower absorption results in higher dosing being needed to maintain an effective concentration in the place of treatment [1]. Researchers have proposed several methods to dominate the low solubility of drugs in water. Drug delivery systems (DDSs) represent a promising approach. The idea is that a drug can be derived or masked in the body to attain the target tissue of action. The proposed system should display low leakage in the overall delivery procedure and release its load at a targeted place. The advanced DDS is designed to release the drug molecules after some chemical factor stimuli, such as the release of environmental pH [2, 3, 4, 5].

Famotidine has a chemical formula of 3‐([2‐(diaminomethyleneamino) thiazol‐4‐yl] methylthio)‐N′‐sulfamoylpropanimidamide. Famotidine is slightly soluble in dehydrated alcohol and water and is highly dissolvable in concentrated acetic acid and diluted mineral acids. Factors such as susceptibility to gastric degradation, poor lipophilicity, and poor aqueous solubility can adversely affect oral bioavailability. Famotidine acts on the parietal cells to inhibit histamine H2‐receptors. This function is associated with a reduction in stomach acid of 90% or more and improves the status of duodenal ulcers. It is also used to treat inflammation of the oesophagus, heartburn, and ulcerations [6, 7].

Zinc sulphide, with the chemical formula ZnS, is an inorganic compound. ZnS has two structural forms, zinc blende and wurtzite crystal. It has a band gap at 340 nm (3.66 eV), and its better chemical stability is used as an optical material for photoluminescent, electroluminescent, and cathodoluminescent devices. The core/shell structure of ZnS quantum dot nanoparticles, with uniform size, good biocompatibility, water‐solubility, low cytotoxicity, and the emission of blue‐green fluorescence, represents a nearly ideal candidate for drug delivery material or carriers with fluorescent labelling [8, 9].

Polymers play a significant role in the surface modification and construction of new adsorbents. In recent years, many different adsorbent and polymer combinations have been applied to drug adsorption and eliminating environmental pollution [10, 11, 12, 13]. To maximize the loading capacity of the therapeutic agent and improve the suspension durability or throughput of the delivery system [14, 15], the surfaces of the ZnS nanoparticles should be modified. The most recent groups used to modify ZnS surfaces are chitosan‐g‐poly (acrylamide) [16], glycopolypeptide [17], polyethyleneglycol [18], cellulose [19], and poly (ethylene glycol) [20]. Polymer coating of ZnS nanoparticles could dramatically increase their dispersion durability and hamper aggregation at physiological pH solution levels compared with corresponding bare materials.

In this research, 3‐aminophenol was grafted onto modified ZnS nanoparticle surfaces with allyl glycidyl ether (AGE). This paper aims to develop a new procedure for drug delivery of famotidine in human simulated enteric fluids using this 3‐aminophenol‐grafted AGE/ZnS nanoparticle as a novel famotidine carrier for sustained release.

## 2 EXPERIMENT

### 2.1 Reagent and instrument

Zinc chloride (ZnCl_2_) and sodium sulphide (Na_2_S), N,N‐dimethylacrylamide, AGE, azobisisobutyronitrile (AIBN), sodium chloride, dimethylformamide (DMF), 3‐aminophenol, acetic acid, hydrochloric acid, and ethanol were purchased from Merck (Darmstadt, Germany). The stock solution (500 mg/L) of famotidine was prepared in deionized water. The pH of the solutions was adjusted using acetate or phosphate buffer.

Infrared absorption spectra were executed on Fourier transform infrared (FTIR) with a Spectrum 100 from the PerkinElmer company (USA). Scanning electron microscope (SEM) images were recorded on an Em‐3200 from the KYKY company (China). Energy‐dispersive X‐ray analysis (EDX) of the sample was conducted with a Mira III with SMAX detector from the Tescan company (Czech Republic). X‐ray diffraction (XRD) analysis was performed with a PW1730 from the Philips company (the Netherlands). Ultraviolet‐visible spectroscopy (UV‐Vis) was carried out on a SERIES 800 from the UVIKON company (Great Britain). Thermogravimetric analysis (TGA) was performed using an sdt851e device from the Toledo company (Mexico). The suspended samples were centrifugated with a universal 320 apparatus from the Hettich Group (Germany). The pH measurements were performed using a pH meter from the Metrohm company (Switzerland).

### 2.2 Synthesis of ZnS nanoparticles

To synthesize the ZnS nanoparticles, 100 ml of 1 M solution of ZnCl_2_ was prepared in deionized water. The 100 ml solution of 1 M Na_2_S was dropped at 50 rpm, stirring at 70°C for 1 h until the white precipitate appeared. The formed precipitate was then filtered and placed in an oven at 120°C for 2 h to dry. Afterwards, 2 g of the precipitate was placed in 50 ml of 0.1 M HCl solution overnight. Then, the obtained precipitate was washed with distilled water until the pH of the precipitate increased to 5, and finally, the precipitate was dried at temperature overnight.

### 2.3 Surface modification of ZnS nanoparticles

To modify the ZnS nanoparticle surface, 2 g of precipitate from the previous step with 20 ml of ethanol, 10 ml of AGE, 2 ml of N, N‐dimethylacrylamide, 0.1 g of purified AIBN were poured into a balloon (50 ml) and then refluxed at 65°C for 7 h in a nitrogen atmosphere.

### 2.4 Grafting of 3‐aminophenol on modified ZnS nanoparticles

**FIGURE 1:**
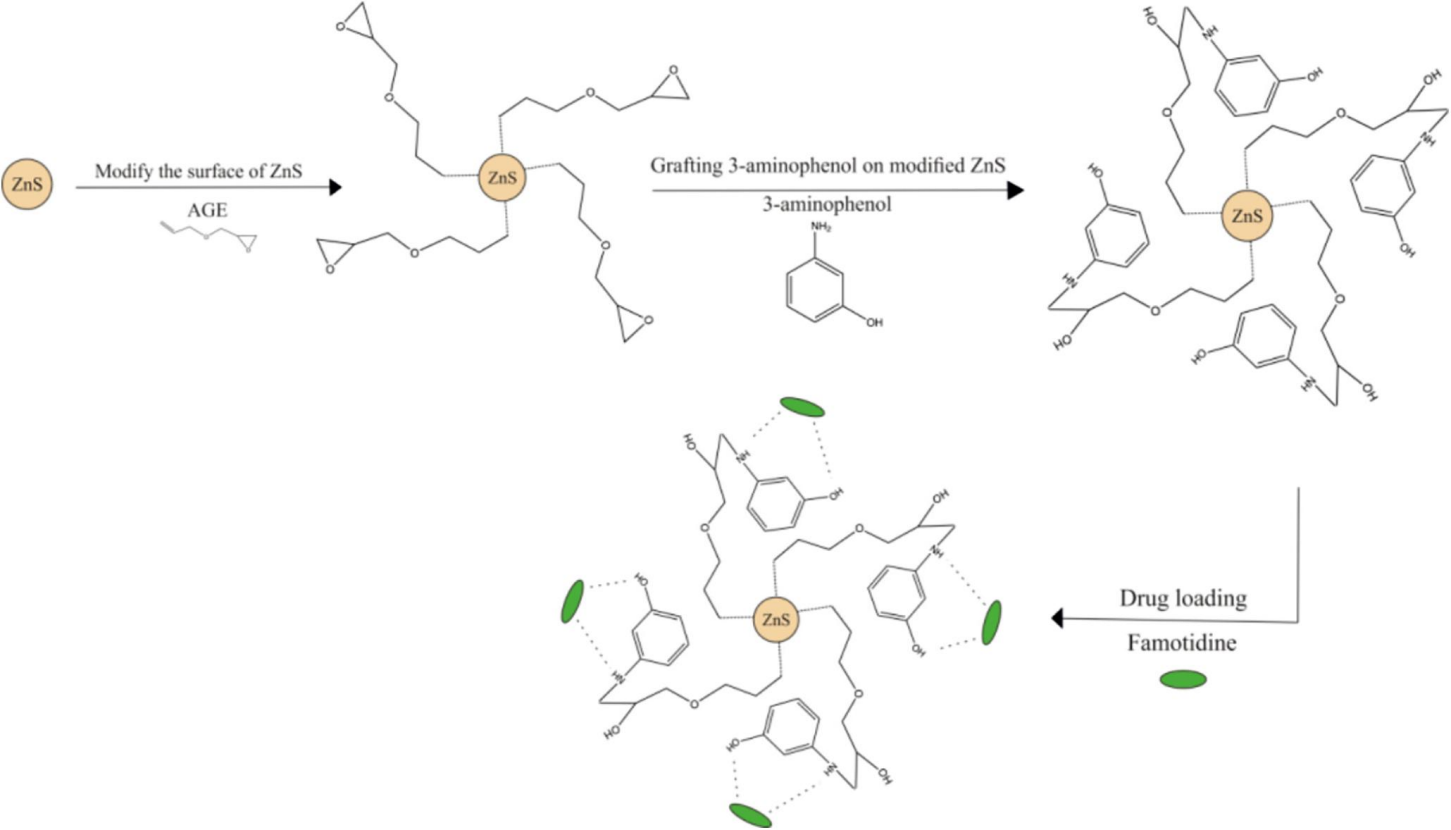
Schematic of synthesis of grafting 3‐aminophenol on modified ZnS and drug loading. ZnS, zinc sulphide

The precipitate obtained from the previous step was filtered and washed with 20 ml ethanol and 10 ml distilled water and then added to a mixture of 40 ml sodium chloride, 10 ml DMF, and 1 g 3‐aminophenol. This mixture was stirred for 2 days at 40°C, the resulting precipitate was washed with the ethanol (20 ml) and distilled water (10 ml) mixture, and the precipitate was then dried at room temperature (Figure 1).

### 2.5 Parameter optimization

For pH optimization, 10 ml solutions of 25 mg/L famotidine were prepared at a pH of 3–7 [21]. The 0.02 g of 3‐aminophenol‐grafted AGE/ZnS was poured into each solution, and the suspensions were stirred for 1 h. The suspensions were then centrifuged, and the solution was analysed by the UV spectrophotometer at 265 nm.

For investigation of the time parameter on the percentage adsorption of famotidine by 3‐aminophenol‐grafted AGE/ZnS, 10 ml solutions of 25 mg/L famotidine were prepared at pH = 5. The 0.02 g of 3‐aminophenol‐grafted AGE/ZnS were poured into the above solutions, and the suspensions were stirred for varying durations. Finally, the suspensions were centrifuged, and the solution was analysed by the UV spectrophotometer at 265 nm.

### 2.6 Adsorption isotherms

The study of isotherm models was performed by adding a constant amount of 3‐aminophenol‐grafted AGE/ZnS (0.02 g) to a series of beakers with 10 ml of a solution (2–100 mg/L) of famotidine in buffer solution (pH 5). The beakers were then stirred at 25°C for 60 min. The amount of famotidine loading in the equilibrium concentration (mg/g) on the 3‐aminophenol‐grafted AGE/ZnS was calculated from the following equation:

(1) $$q_{e}=\frac{(c_{o}{-c}_{e})V}{m}$$

where C_0_ is the initial concentration of famotidine (mg/L), and Ce is the concentration of famotidine in equilibrium (mg/L), V is the volume of the test solution (L), and m is the mass of the 3‐aminophenol‐grafted AGE/ZnS (g) [22].

### 2.7 In vitro famotidine release

The release profiles of famotidine from 3‐aminophenol‐grafted AGE/ZnS were analysed in a simulated gastric (pH = 1.2) and simulated intestinal (pH = 7.4) environments. Famotidine‐loaded 3‐aminophenol‐gra/fted AGE/ZnS (0.1 g) was placed in beakers at 37°C, and the 50 ml release media (0.1 M of HCl/phosphate buffer) was added. These samples were stirred for 1 h (pH = 1.2) and 30 h (pH = 7.4) at 50 rpm. Sampling was taken at scheduled intervals, and the famotidine content of each sample was assessed by UV‐Vis [23].

### 2.8 Kinetic modelling of famotidine release

To study the mechanism of famotidine‐release process initiation, several kinetic patterns have been investigated for fitting experimental releasing data with mathematical patterns. These models include zero‐order (Equation (2)), first‐order (Equation (3)), Korsmeyer–Peppas (Equation (4)), Hixon–Crowell (Equation (5)), and Higuchi (Equation (6)) mathematical patterns:

(2) Qt=k0t

(3) ln Qt=ln Q0+k1t

(4) $$\text{Qt}\;=\;K{\text{KPt}}^{n}$$

(5) $${\text{Q0}}^{1/3}{‐Q1}^{1/3}\;=\;\text{KHCt}$$

(6) $$\text{Qt}\;=\;\text{KH}\sqrt{t}$$

where Q_t_ is a fraction of the famotidine released at t (time), Q_0_ is the initial amount of famotidine, K_0_, K_1_, K_KP_, K_HC_, and K_H_ are the kinetic constants, and n is the value of the diffusion exponent characteristic of the famotidine‐release mechanism [24].

## 3 RESULTS AND DISCUSSION

### 3.1 Characterization

**FIGURE 2:**
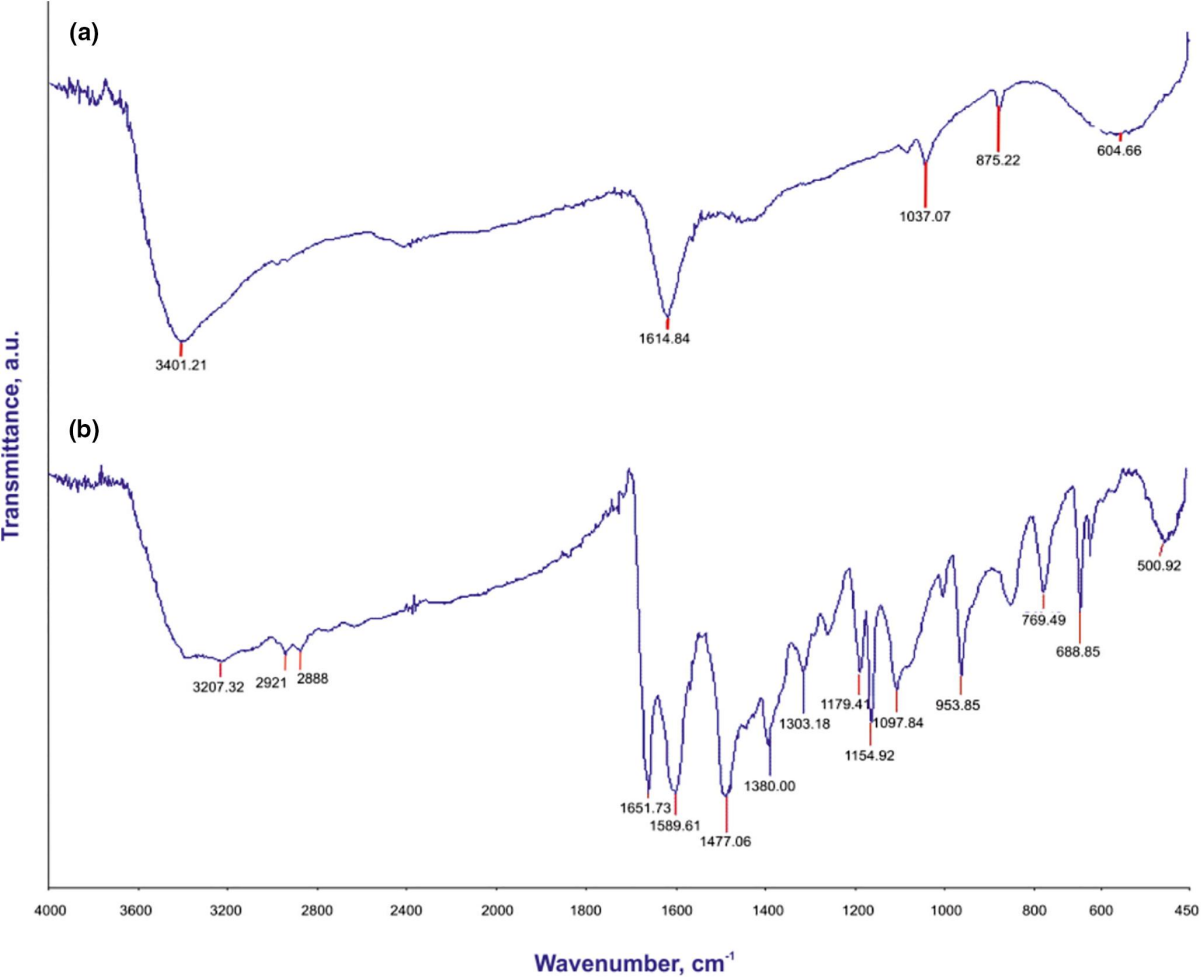
Fourier transform infrared spectra of (a) zinc sulphide nanoparticles, (b) 3‐aminophenol‐grafted allyl glycidyl ether/zinc sulphide

The FTIR spectra of all stages of the adsorbent were obtained and are shown in Figure 2. From the first step (ZnS nanoparticles), the obtained spectra in Figure 2a display a peak at 3401 cm^−1^ wavelengths attributed to the stretching vibration of O‐H from adsorbed water, and a peak at 604 cm^−1^ is ascribed to the ZnS band (i.e. relating to sulphides) [25]. In the second stage (Figure 2b), 3‐aminophenol on modified ZnS nanoparticles with AGE, a broad absorption band is witnessed at 3207 cm^−1^ related to the O‐H group, a peak at 2921 cm^−1^ is associated with aliphatic stretching vibrations of C‐H, and a peak at 688 cm^−1^, is assigned to the vibration of N‐H of the amine group. The band at 1155 cm^−1^ corresponds to the stretching vibration of C‐O. In addition, two peaks at 1477 and 1589 and a peak at 1651 cm^−1^ are ascribed to the stretching vibrations of C‐C and C = C from an aromatic ring, respectively [26].

**FIGURE 3:**
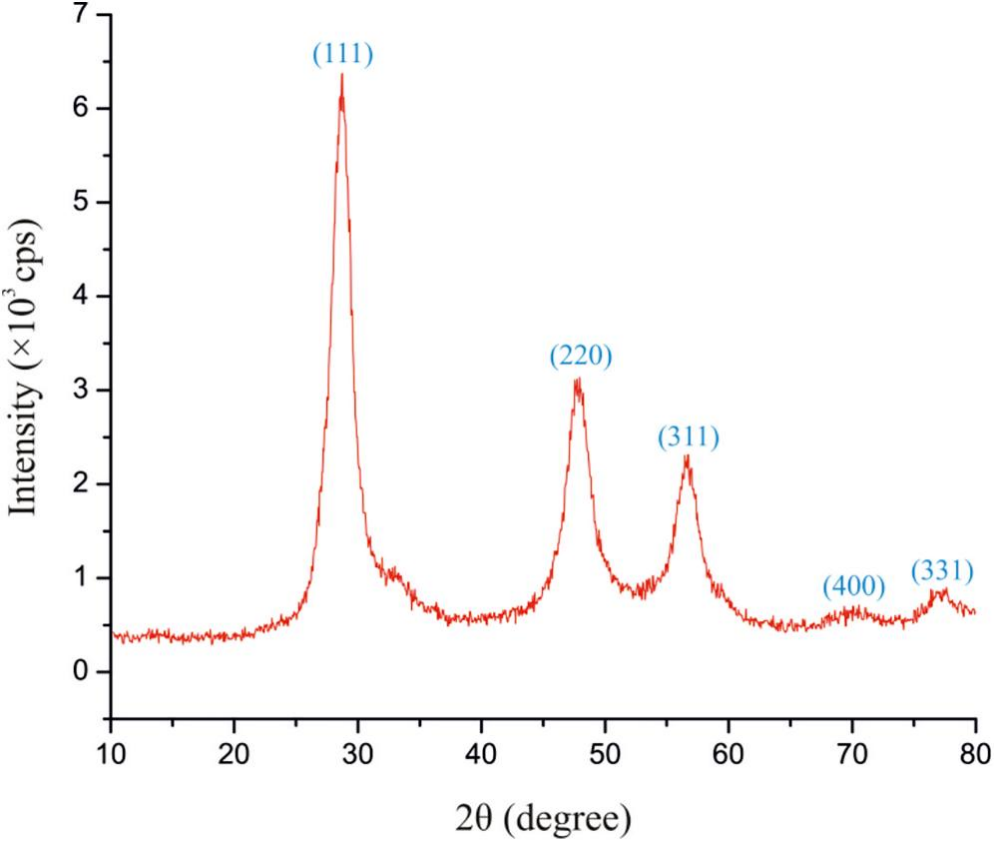
X‐ray diffraction analysis of 3‐aminophenol‐grafted allyl glycidyl ether/zinc sulphide sample

Figure 3 displays the XRD analysis of 3‐aminophenol‐grafted AGE/ZnS nanoparticles. The XRD data of 3‐aminophenol‐grafted AGE/ZnS nanoparticles exhibit the zinc blende structure with cubic phase. The crystal planes of (111), (220), and (311) of zinc blende structure were obtained at three broad bands with 2θ values of (28.75), (48.17), and (56.69), respectively [1, 27].

**FIGURE 4:**
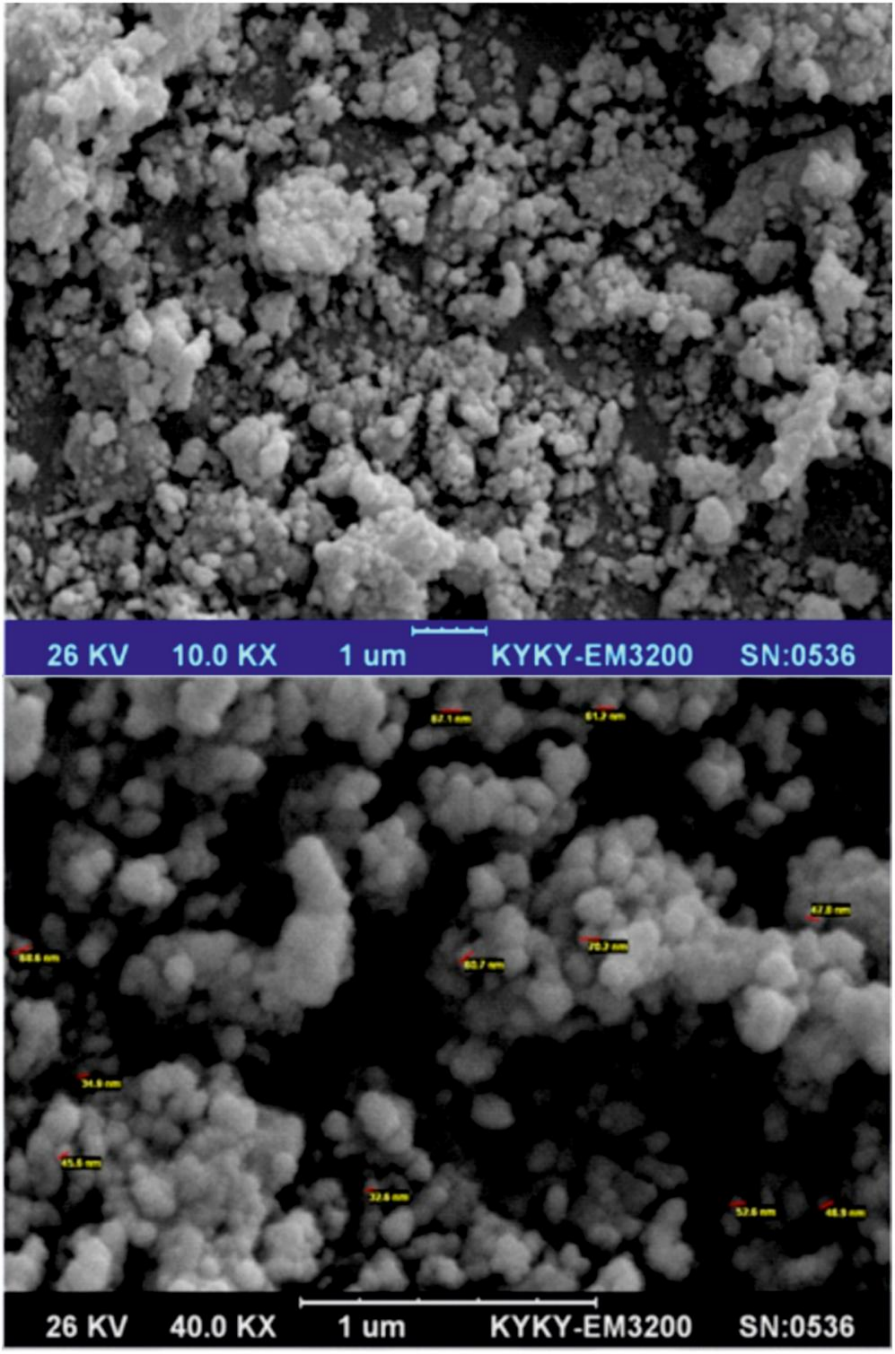
Scanning electron microscope images of 3‐aminophenol‐grafted allyl glycidyl ether/zinc sulphide

Figure 4 shows the SEM images of the 3‐aminophenol‐grafted AGE/ZnS nanoparticles. These images indicate that the 3‐aminophenol‐grafted AGE/ZnS is spherical with a mean diameter of 30–80 nm. This figure also shows that the surface of the 3‐aminophenol‐grafted AGE/ZnS nanoparticles is uneven and has pores at a nanometre scale. These pores are effective in increasing famotidine adsorption.

**FIGURE 5:**
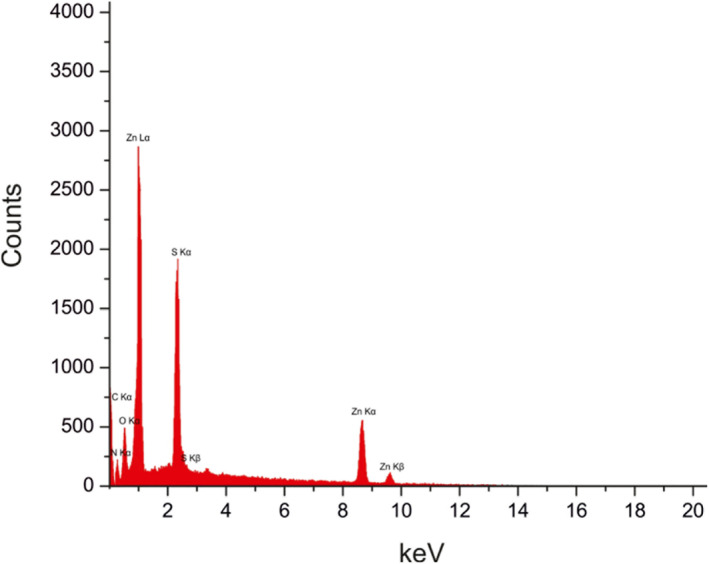
Energy‐dispersive X‐ray spectra of 3‐amitpdelnophenol‐grafted allyl glycidyl ether/zinc sulphide

Figure 5 explains the EDX analysis of 3‐aminophenol‐grafted AGE/ZnS. It is found that zinc, sulphur, carbon, nitrogen, and oxygen are the main identified elements, implying that the obtained nanosorbent is composed of ZnS, AGE, and 3‐aminophenol.

**FIGURE 6:**
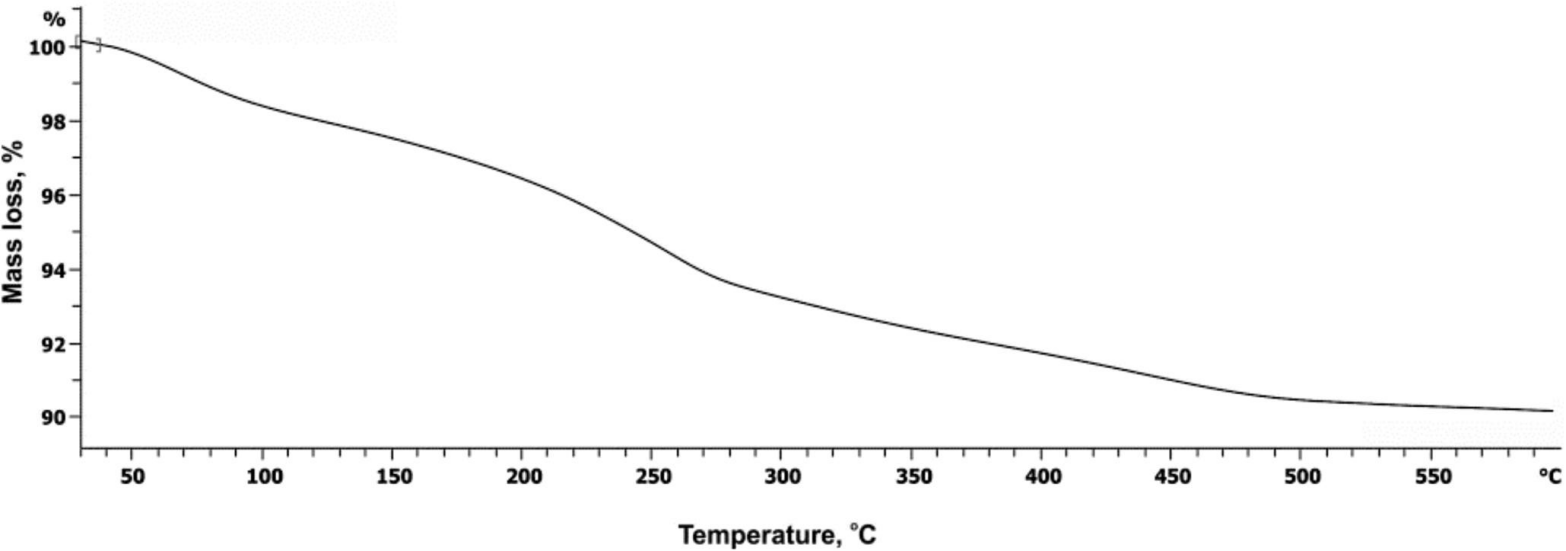
Thermogravimetric analysis curve of 3‐aminophenol‐grafted allyl glycidyl ether/zinc sulphide

To obtain the information of surface‐bonded organic groups, a thermogravimetric analysis was conducted. Figure 6 shows the TGA curve of 3‐aminophenol‐grafted AGE/ZnS. The mass decrease to around 100°C corresponds to the thermodesorption of water on the surface. As the temperature rises above 250°C, the polymer starts to decompose and is thoroughly decomposed at high temperatures. The total mass loss of 3‐aminophenol‐grafted AGE onto ZnS was 7.5%.

### 3.2 Optimization of drug loading parameters

**FIGURE 7:**
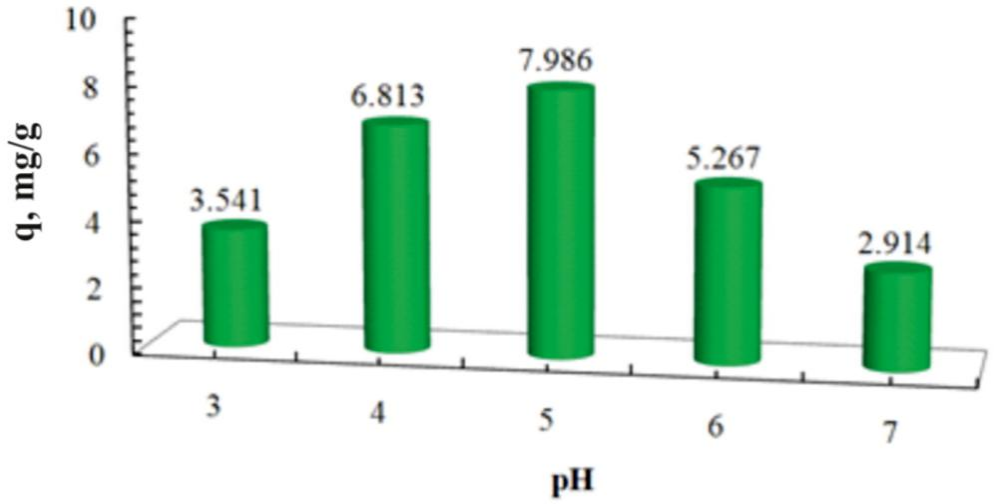
Sorption of famotidine using adsorbent at different pH

Maximum sorption of famotidine at different pH values (3–7) was performed by the batch method. As shown in Figure 7, max sorption occurs at pH = 5. This reflects the most probable possibility of forming a hydrogen bond at mildly acidic pH. Hydrogen bonding can be the main adsorption affinity in the mechanism of famotidine adsorption on 3‐aminophenol‐grafted AGE/ZnS [28]. The hydroxyl group of 3‐aminophenol connects with an amino group of famotidine via a hydrogen bond and provides maximum affinity (Figure 1).

**FIGURE 8:**
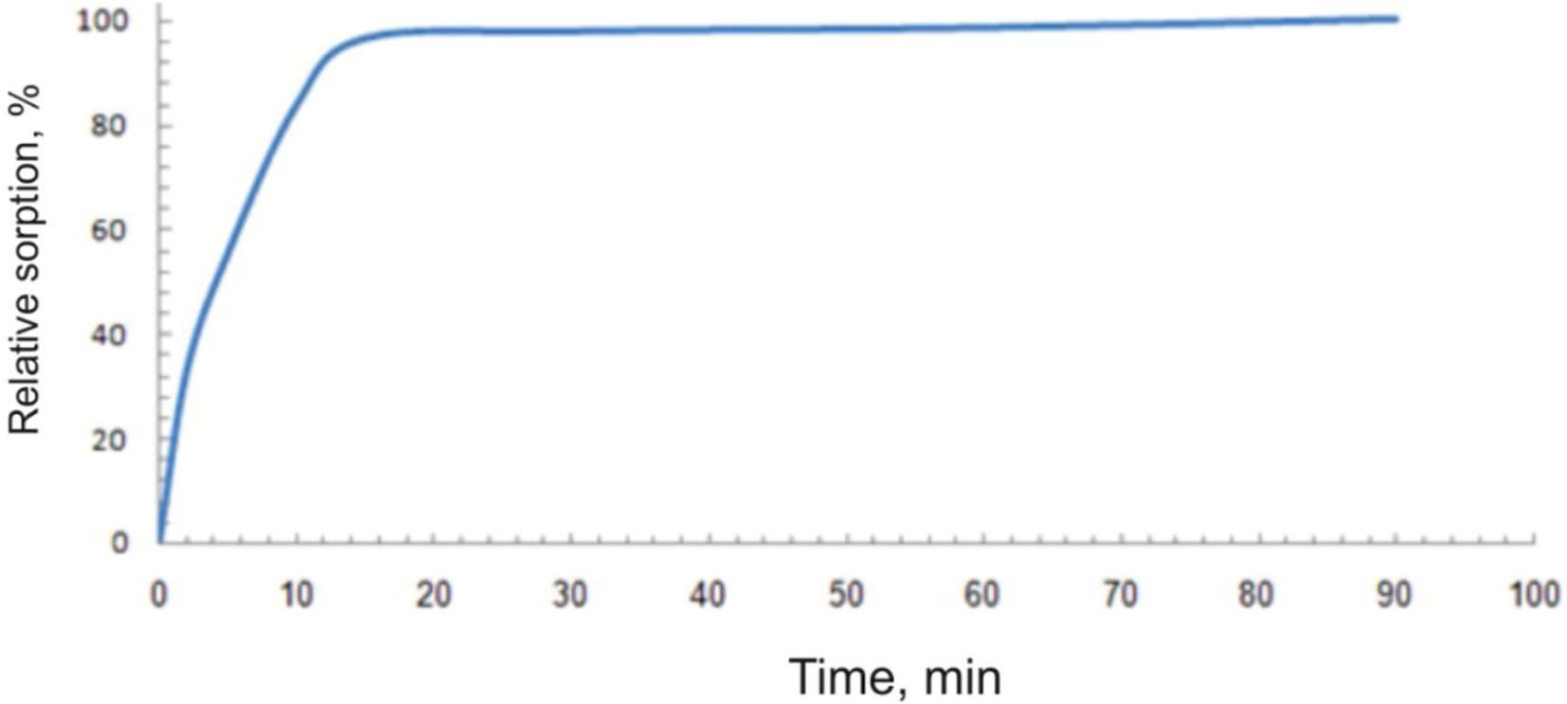
Kinetics of sorption of the drug onto 3‐aminophenol‐grafted allyl glycidyl ether/zinc sulphide

Alkaline pH (pH > 7) was not studied, because in alkaline pH, famotidine was decomposed [29]. The kinetic of famotidine sorption at pH = 5 can be seen in Figure 8 and Table 1. Concerning the adsorption rate result, the famotidine shows fast adsorption by the adsorbent such that within the first 5 min, 55% of famotidine was adsorbed. Only 60 min are required for 98% saturation sorption of famotidine.

**TABLE 1:** Kinetic data of famotidine sorption

First‐order kinetic		Second‐order kinetic	
q_e_ (mg g^−1^)	5.48	q_e_ (mg g^−1^)	10.41
k_1_ (min^−1^)	0.074	k_2_ (g mg^−1^ min^−1^)	0.023
R^2^	0.89	R^2^	0.99

The results obtained from the famotidine adsorption kinetics by 3‐aminophenol‐grafted AGE/ZnS in pseudo‐first order and pseudo‐second order show that famotidine adsorption follows from the adsorption kinetic of the pseudo‐second‐order at R^2^ = 0.999 (Table 1) [3]. The rapid adsorption of drug molecules was attributed to the availability of active sites in 3‐aminophenol‐grafted AGE/ZnS.

### 3.3 Adsorption isotherms

Adsorption was investigated of the exhibition of sorbed famotidine per nanoparticle of 3‐aminophenol‐grafted AGE/ZnS, and adsorption isotherms were evaluated as a function of equilibrium concentration at room temperature (25°C) for optimized famotidine adsorption. The Langmuir isotherm model is valid for monolayer adsorption on a surface with several identical active sites. The Langmuir model supposes that adsorption energies are uniform across the modified surface, and adsorbates have no transmigration in the surface plane. The Langmuir equation is shown as [30]

(7) $$\frac{c_{e}}{q_{e}}=\frac{c_{e}}{q_{m}}+\frac{1}{q_{m}K_{L}}$$

where q_m_ is the maximum sorption of famotidine per gram of 3‐aminophenol‐grafted AGE/ZnS (mg g^−1^), and K_L_ is the Langmuir constant (L mg^−1^). In addition, the dimensionless separation factor (R_L_) as an essential characteristic of this model can be expressed in the following relation:

(8) $$R_{L}=\frac{1}{1+\left(K_{L}c_{0}\right)}$$

The literature study [31] ascertained that the value of R_L_ shows that the sorption nature is irreversible (R_L_ = 0), desirable (0 < R_L_ < 1), linear (R_L_ = 1), or undesirable (R_L_ = 1). Table 1 shows R_L_ in a 0–1 range with a value of (0.483), which confirms the desirable sorption of famotidine. The next model, by Freundlich, is an empirical model isotherm employed to describe heterogeneous systems distinguished by an empirical equation with heterogeneity factor (1/n) [32]:

(9) $${\text{ln q}}_{e}\;=\;{\text{ln K}}_{F}+\frac{1}{n}{\text{ln c}}_{e}$$

where K_F_ is the Freundlich constant (L g^−1^), n is the intensity of sorption, and (1/n) is the inhomogeneous factor and represented by three values: (1/n > 1) sorption is unfavourable, (1/n = 0) sorption is irreversible, and (0 < 1/n < 1) sorption is favourable. The empirical evidence of the Freundlich model corresponds to sorption on a modified heterogeneous surface. The Freundlich model assumes that the enthalpy of sorption logarithmically decreases with increasing fractions of occupied active sites.

The Temkin model suggests the sorption energy linearly decreases when the level of occupancy of the sorptional centres of 3‐aminophenol‐grafted AGE/ZnS increases [33]:

(10) $$q_{e}=\frac{\text{RT}}{b}\text{ln A}+\frac{\text{RT}}{b}{\text{ln c}}_{e}$$

where RT/b is the Temkin constant (B) indexed to the heat of adsorbate sorption (J mol^−1^), A is the Temkin constant of binding equilibrium (L g^−1^), R (8.314) is the gas constant (J mol^−1^ K^−1^), and T is the absolute temperature (K).

The parameters of adsorption isotherms summarized in Table 1 demonstrate that the data are fitted well in the Freundlich isotherm model with R^2^ = 0.969. This result of the Freundlich isotherm confirms that famotidine molecules are heterogeneously sorbed onto the 3‐aminophenol‐grafted AGE/ZnS. Furthermore, concerning the value of R^2^ = 0.928 for the Temkin isotherm, the next approach for famotidine adsorption with this model was carried out on the 3‐aminophenol‐grafted AGE/ZnS.

### 3.4 Drug release

**FIGURE 9:**
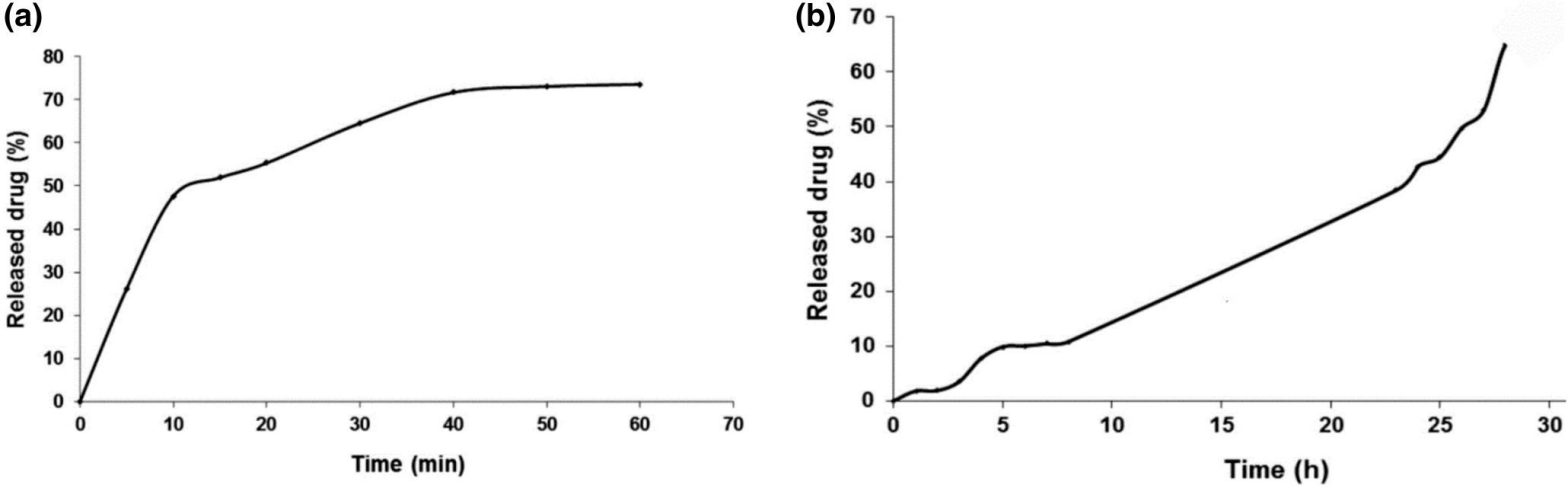
Famotidine release profiles (a) simulated gastric fluid, 37°C and pH = 1.2. (b) simulated intestinal fluid, 37°C and pH = 7.4

The release of famotidine drug by 3‐aminophenol‐grafted AGE/ZnS in simulated gastric fluid with a pH of 1.2 is displayed in Figure 9a, and simulated intestinal fluid with a pH of 7.4 is displayed in Figure 9b. Nearly 65% of the drug was released in the simulated intestinal environment at pH = 7.4 for 30 h. In the early hours, 3‐aminophenol‐grafted AGE/ZnS released 10% of the drug, and then the release of famotidine with gentle slope was increased until 40%. This low release can be due to famotidine molecules adsorbed in the ZnS pores that are not grafted with the polymer. Over time, an equilibrium is created between the famotidine molecules and the adsorbent surface, which indicates that the amount of famotidine in the media is constant [1, 34]. Famotidine, with its alkaline properties onto 3‐aminophenol‐grafted AGE/ZnS, is most stable in phosphate buffer solutions, whereas in the simulated gastric environment with harsh acidic medium, famotidine was released more quickly. Approximately 73% of the drug was released in the gastric environment for 60 min; after that, the trend of drug‐releasing from 3‐aminophenol‐grafted AGE/ZnS was constant for 10 min, and then the release of famotidine was gradually decreased. The acidic pH of the simulated gastric solution results in the dissolution of adsorbent and subsequently a faster release rate.

### 3.5 Kinetic model study for famotidine‐releasing process

In this work, the experimental release data for 3‐aminophenol‐grafted AGE/ZnS were analysed by various models to realize the release process of famotidine from the uneven surface of the carrier. The calculated release parameters of zero and first‐order, Higuchi, Korsmeyer–Peppas, and Hixon–Crowell mathematical patterns are reported in Table 2. The results of the Korsmeyer–Peppas models demonstrated a high fitting coefficient value (R^2^) for the famotidine‐releasing process. The Korsmeyer–Peppas model values for the release exponent (n < 0.5) of famotidine suggest a controlled diffusion mechanism for famotidine release with a quasi‐Fickian process [35, 36].

**TABLE 2:** Isotherm parameters of famotidine adsorption

Langmuir isotherm model			
qm (mg g^‐1^)	KL (L mg^‐1^)	RL	RL
41.667	0.036	0.483	0.925
Freundlich isotherm model			
Kf (L g^‐1^)	n		R^2^
1.490	1.248		0.969
Temkin isotherm model			
A (L g^‐1^)	B (J mol^‐1^)	b (J mol^‐1^)	R^2^
1.071	5.971	414.939	0.928

### 3.6 Comparison of adsorbent used in current work with other reported adsorbents

The performance of 3‐aminophenol‐grafted AGE/ZnS used for adsorption of famotidine in the present work was compared with other reported adsorbents. As is shown in Table 3, the contact time (60 min), pH (5), R^2^ (0.969), maximum sorption (41.667 mg/g), and percentage of famotidine released in acidic and phosphate media are preferable for 3‐aminophenol‐grafted AGE/ZnS in comparison with other adsorbents [37, 38, 39, 40, 41, 42]. (Table 4).

**TABLE 3:** Kinetic models of famotidine release from 3‐aminophenol‐grafted AGE/ZnS

Simulated Gastric Fluid (pH = 1.2)										
Zero‐order		First‐order		Higuchi		Korsmeyer–Peppas			Hixon–Crowell	
K_0_	R^2^	K_1_	R^2^	K_H_	R^2^	K_KP_	n	R^2^	K_HC_	R^2^
0.73	0.79	0.014	0.67	0.5	0.64	17.13	0.38	0.90	0.017	0.71

Simulated Gastric Fluid (pH = 7.4)										
Zero‐order		First‐order		Higuchi		Korsmeyer–Peppas			Hixon–Crowell	
K_0_	R^2^	K_1_	R^2^	K_H_	R^2^	K_KP_	n	R^2^	K_HC_	R^2^
1.90	0.56	0.03	0.45	0.5	0.44	26.2	0.44	0.75	0.041	0.50

Abbreviations: AGE, allyl glycidyl ether; ZnS, zinc sulphide.

**TABLE 4:** Comparison of famotidine adsorption by various adsorbents

Adsorbent	Time	pH	Freundlich(R^2^)	Maximum sorption (q_m_)	% release		References
					Acidic	Phosphate	
					Media	Media	
3‐Aminophenol‐grafted on modified ZnS nanoparticles	60 min	5	0.969	41.66	73% (60 min)	65% (30 h)	This work
Microcrystalline Cellulose	24 h	7	0.996	3.52	99.6% (15 min)	─	[37]
Carboxylic‐modified mesoporous silica	4 h	7	0.998	155.52	98% (2 h)	80% (6 h)	[38]
Magnetic nanoparticles modified	10 min	5	0.9553	15.20	86% (60 min)	60% (30 h)	[39]
By iminodiacetic acid							
Poly[N‐isopropylacrylamide‐co‐allyl glycidyl/iminodiacetic] onto iron oxide nanoparticles modified using 3‐mercaptopropyltrimethoxysilane	5 min	7	0.9885	116.30	73% (60 min)	70% (30 h)	[40]
Activated Alginate‐Graphene oxide Beads	24 h	7	0.7491	35.50	20% (24 h)	─	[41]
Activated carbon	21 days	5.6	0.9764	86.20	Deionized water media		[42]
					5% (4 days)		

## 4 CONCLUSIONS

A novel nanosorbent based on ZnS nanoparticles was successfully prepared. This research presented showed that desorption of famotidine onto 3‐aminophenol‐grafted AGE/ZnS had sustained released in simulated intestinal and gastric fluids. Approximately 65% of famotidine was released in the simulated intestinal environment at pH = 7.4 for 30 h. The results reveal that the presented technique has benefits, including facileness, rapidity, and low nanosorbent utilization. The maximum sorption, with a value of 41.667 mg/g, was excellent. According to the study of isotherms, the Freundlich model is consistent for adsorption of famotidine. The famotidine release kinetics of the 3‐aminophenol‐grafted AGE/ZnS were fitted to the Korsmeyer–Peppas model and quasi‐Fickian diffusion. In conclusion, the 3‐aminophenol‐grafted AGE/ZnS was highly capable of adsorption and desorption of famotidine drugs.
